# Insect as feed ingredients for pigs

**DOI:** 10.5713/ab.21.0475

**Published:** 2022-02-01

**Authors:** Jinsu Hong, Yoo Yong Kim

**Affiliations:** 1 Department of Animal Science, South Dakota State University, Brookings, SD 57007, USA; 2 Department of Agricultural Biotechnology, and Research Institute of Agriculture and Life Sciences, Seoul National University, Seoul 08826, Korea

**Keywords:** Alternative Feed Ingredient, Insects, Insect Meal, Pig

## Abstract

Among edible insects, black soldier fly (*Hermetia illucens*), yellow mealworm (*Tenebrio molitor*), and common housefly (*Musca domestica*) have been considered as an alternative protein source for pigs. Because they are easy to breed and grow in the organic wastes, and they have well-balanced nutritional value as a protein source for pigs. The black soldier fly larvae and mealworm could replace the fish meal in the diets for weaned pigs without adverse effects on growth performance and nutrient digestibility. Black soldier fly could also be included in the finishing pig’s diet without any negative effects on the growth performance and pork quality of the market pigs. Insect products showed a greater standardized ileal digestibility value of amino acids than conventional animal proteins in growing pigs. Due to the limited amount of insect products used for pig feeding study, most previous pig studies have been conducted in weaned pigs. Thus, further study is needed about the optimal inclusion level of insect products in every phase diet from weaned pigs to sows. The use of insect products in swine diets has some challenges in terms of cost, supply, and safety. Lastly, intrinsic differences among insect species, processing method, and feeding phase should be taken into consideration for the use of insect products in the swine diets.

## INTRODUCTION

As the world population is expected to be more than 10 billion by the year 2050, consumption of chicken and pork is also expected to increase from 2010 to 2050 years by 173% and 105%, respectively [1,2]. The Food and Agriculture Organization (FAO) recommended finding alternatives to conventional animal feed ingredients to reduce competition between humans and animals for limited food chain resources [1,3]. In this global movement, world market value for insects has increased rapidly by 20% to 30% annually with the trends of finding novel alternatives to conventional feed ingredients. The biggest market for edible insects is Asia-Pacific countries, but there is no legislation on the use of insects as feed so far. On the other hand, black soldier fly (BSF; *Hermetia illucens*) was allowed to be used as an ingredient for aquaculture feed in the United States, and the use of BSF was authorized for poultry and aquaculture feed in Canada. Recently, the European Union passed legislation that authorizes the use of some processed animal protein and insect meals in feeds for poultry and pigs. As insects become more important as an alternative ingredient in animal feeds, their value will also increase. Therefore, this review aims to report and discuss the previous swine research on the use of insects and the critical points to consider for the use of insects in swine feeds.

## INSECTS AS A FEED INGREDIENT

More than 1,900 species of edible insects have been identified, and insect research has been conducted to find a food resource [4]. Unfortunately, consumers have more negative perceptions of edible insect consumption directly as insect-containing food compared to indirect consumption of insects through meat produced by insect-containing feeds [5]. On the other hand, insects are considered a good protein or fat source for animals with high digestibility and attractive flavor. In the perspective of sustainability, insects grow very fast with a short life cycle, and they can be fed organic wastes, including food waste, manure, garbage, or plastics [6]. Also, insects emit fewer greenhouse gases and ammonia and have a less carbon footprint than other livestock animals [7,8]. Additionally, the feed conversion efficiency and protein production efficiency for insects are greater than pigs and cattle [4,9,10]. Therefore, insects have a great potential to be used as a sustainable protein source for livestock animals feed to replace feed ingredients, including soybean meal (SBM) and fish meal.

The insects for animal feed ingredients are usually processed by slaughtering (heating or freezing) and post-slaughtering (drying, grinding, extraction, or hydrolyzation) procedures [11]. The insects are cleaned and dried through blanching, freezing, chilling, and drying to prevent microbial growth during long-term storage and transport. After the slaughtering process, the insects are dried to have 4% to 5% moisture content to avoid nutrient degradation and microbiological spoilage [12,13]. The dried insects are usually finely ground to similar particle size to other feed ingredients or subjected to oil extraction or hydrolysis [14]. Furthermore, functional substances such as chitin and chitosan also can be extracted from the insects [15,16].

## SWINE RESEARCH WITH INSECT PRODUCTS

Insects studied as most promising for industrial production and animal feed ingredient in the world are the BSF (*Hermetia illucens*), yellow mealworm (*Tenebrio molitor*, TM), and common house fly (HF; *Musca domestica*). Because these insect species can grow on livestock manure or organic waste with good feed conversion efficiency [6,17], they are considered sustainable feed ingredients for livestock animals. Most previous studies evaluating the nutritional value of edible insects or insect meals have been conducted for the poultry diet. This is because the amounts of insects required for a poultry experiment is much lower than that for a swine experiment. The size of the insect-rearing companies or growers is relatively too small to provide sufficient insect products for swine feed. Moreover, the price of insect protein products is still high compared to the price of commonly used protein ingredients, including SBM and fish meal. Therefore, there is relatively limited information about the nutritional value of insect meal or insect product in pigs compared to poultry (broilers and laying hens).

The swine research with insect species and countries from 2000 to 2021 years are presented in Table 1. Recently, some swine research for BSF larvae was conducted in the diets for suckling pigs [18] and growing pigs [19] in Africa countries. Asia, especially China and South Korea, is the major region conducting swine research to evaluate the nutritional value of insect products in swine diets. In China, various insect species, including BSF, HF, mealworm, or superworm, have been studied in different feeding phases, from weaned pigs to finishing pigs [20–23]. However, the study with growing pigs was for ileal digestibility determination [23], and the study with finishing pigs was for microbiota and gene expression analyses [21]. In South Korea, mealworms (TM larvae and *Ptecticus tenebrifer* larvae) have been studied as an alternative protein source for conventional protein ingredients, including SBM and fish meal in the diets of weaned pigs [24–26]. Moreover, there were two studies evaluating the ileal digestible value of TM larvae products, including dried TM larvae [27], defatted TM larvae meal [28], and hydrolysate of TM larvae [28] in growing pigs. In Europe, swine research on BSF larvae as a protein ingredient for the swine diet has been conducted actively [29–35]. Among the swine studies, Altmann et al [31] and Chia et al [35] have investigated the supplemental effects of BSF larvae on growth performance and carcass characteristics of finishing pigs. On the other hand, there are some growing pig studies for investigating the dietary effects of TM larvae on the metabolic response [36], antioxidant or stress response [37], and microbiota response [34]. In North America, such as Canada and United States, animal research has been mainly conducted on BSF larvae. The full-fat BSF larvae meal was used for feeding trial study with growth performance and immune response of weaned pigs [38]. The full-fat BSF larvae meal and defatted BSF larvae meal were used for evaluating the standardized ileal digestibility (SID) in growing pigs [39]. Unfortunately, it was hard to find any published articles or data from Latin American countries conducting swine research with those insect species (BSF, HF, and mealworm). Considering that articles reviewed on utilization of the insect products in poultry diets have been published in Brazil [40], it is anticipated that small- or large-scale pig research with insect products has been conducted; the data from swine studies may not be published yet.

### Black soldier fly (*Hermetia illucens*)

The BSF larvae or prepupae products have a good quality of amino acid (AA) profile compared to the SBM and other animal proteins. The nutritional value of BSF larvae products used in the swine research is presented in Table 2. Most of previous swine studies used dried BSF larvae meal (dried full-fat BSF larvae meal) containing average 42% of crude protein (CP) ranged from 35.9% to 48.1% and average 42.5% of ether extract (EE) ranged from 36.8% to 48.1% [18,19,21, 29,38]. Partially defatted BSF larvae meal showed an average of 59% CP and 9% EE contents [32], and defatted BSF larvae meal showed an average of 51% CP and 10% EE contents [29,38]. Crosbie et al [38] reported that the SID values of Lys, Met, and Thr for full-fat BSF larvae meal were similar to those for SBM and the SID value of Arg for full-fat BSF larvae meal was less than that for SBM. Also, the SID values of Lys, Thr, and Val for defatted BSF larvae meal were similar to those for SBM, whereas the SID value of Met for defatted BSF larvae meal was less than that for SBM.

In weaning pigs, 50% of animal proteins, including fish meal, spray-dried blood meal, and blood plasma replaced with full-fat BSF larvae meal (d 0 to 7, 14.76%; d 7 to 21, 7.5%; d 21 to 42, 1%) had no impact on the final body weight (BW, d 42) and average daily gain (ADG) for the overall period (d 0 to 42), whereas it improved gain to feed (G:F) ratio for d 7 to 21 by 12% without any effects on weights of liver and gastrointestinal tract and histomorphology for jejunum and ileum at 8 d postweaning [39]. Inclusion of partially defatted BSF larvae meal at 10% to replace SBM increased average daily feed intake (ADFI) of weaned pigs for d 24 to 61 due to increased diet palatability [32]. Also, supplementation of 10% partially defatted BSF larvae meal had no adverse influences on blood profile, nutrient digestibility, small intestinal morphology, or histological features. Moreover, weaned pigs fed the diet with 8% full-fat BSF prepupae (EE, 41%) or 5.42% defatted BSF prepupae (EE, 8%) showed no influence on their growth performance, villus height, and crypt depth for jejunum, and counts of *Lactobacilli* and *D-Streptococci* compared to the pigs fed the control diet with toasted soybeans [29].

In growing pigs, Nekrasov et al [30] reported that supplementation of 0.8% BSF larvae did not affect the growth performance of the growing pigs with higher blood protein concentration and blood leukocytes counts compared to the control diet. Also, Chia et al [19] reported that 100% replacement of fishmeal (10%) with BSF larvae meal (18.5%) in growing pigs’ diet had no difference on the growth performance, red blood cell indices, and white blood cell. In finishing pigs, Chia et al [35] reported that increasing level of BSF larvae meal (9% to 14%) in finishing pigs’ diet to replace 50% to 100% of fish meal increased final BW, ADG, and feed conversion ratio for the overall period, resulted in an increase of carcass weight of finishing pigs. Altmann et al [31] also indicated that replacement of SBM with partly defatted BSF larvae meal (50% or 70% for 25 to 75 kg of BW and 100% for 75 to market weight) did not affect carcass weight, instrumental tenderness, cooking loss, and 2-thiobarbituric acid reactive substances value for *longissimus* muscle. Also, they found that supplementation of partly defatted BSF larvae meal in finishing pigs’ diet improved overall flavor and juiciness of pork loin by sensory analysis. Collectively, BSF larvae products could be included in the diets for weaned pigs to finishing pigs as an alternative protein source. However, there is a need for further study to investigate the optimal inclusion level of BSF larvae products in the swine diets and potential effects on the porky quality perceived by consumers.

In terms of gut microbiota, weaned pigs fed the diet containing BSF larvae meal (10%) showed a good influence on cecal microbiota with short-chain fatty acids-producing bacteria when compared to the control diet [33]. Furthermore, dietary BSF larvae meal up to 10% positively affected the gut maturity by small intestinal mucin composition without any adverse effects on the histological score in the gut of the piglets. Similarly, Kar et al [34] reported that dietary BSF larvae meal induced a more enriched small intestinal microbiome of growing pigs compared to dietary SBM in regard to a healthy gut status in pigs. In addition, dietary BSF larvae meal did not cause a systemic inflammatory response such as pro- and anti-inflammatory cytokines and chemokines. Yu et al [21] demonstrated that supplementation of 4% BSF larvae meal had a positive influence on the colonic microbiota of finishing pigs with an increased abundance of *Lactobacillus* and butyrate-producing bacteria, resulting in an increase of short-chain fatty acids concentrations in colonic digesta. Furthermore, BSF larvae supplementation at 4% up-regulated the colonic mucosal gene expression of anti-inflammatory cytokine (interleukin 10) and intestinal barrier function (zonula occludens-1, occludin, and mucin-1) compared with the control group. Therefore, BSF products could be utilized as a sustainable protein source for swine diets, and the functional effects of dietary BSF products should be investigated in the future.

### Mealworm (*Tenebrio molitor* larvae)

The TM larvae, known as yellow mealworms, are considered a good protein and fat sources for animal feed [17]. The TM larvae can be produced easily with a stable protein content [42], and their chemical composition and growth efficiency can be influenced by the ingredients and nutritional composition of their diets [43,44].

The nutritional value of TM larvae products used in the swine research is presented in Table 3. Most of previous swine studies used dried TM larvae meal (dried full-fat TM larvae meal) containing average 55% of CP ranged from 46.3% to 74.0% and average 27% of EE ranged from 10.3% to 34.6% [24,26,27,28,36]. Cho et al [28] used defatted TM larvae meal and hydrolysate of TM larvae products to investigate the SID values of AA for those products. The defatted TM larvae meal contained 68% of CP and 4% of EE, and the hydrolysate of TM larvae contained 58.9% and 0.3% of EE with higher AA contents than the general TM larvae meal.

The supplemental effects of TM larvae products in swine diets have been investigated from the perspective of alternative protein sources or functional properties. In weaning pigs, the inclusion of TM larvae meal from 0% to 6% linearly increased BW and ADG of pigs [24]. It also tended to increase the ADFI and G:F ratio of pigs linearly over a period of 5 weeks. Also, they reported that the increase of TM larvae in weaned pigs’ diet improved utilization of protein or nitrogen (N) in weaned pigs as evidenced by tendencies of increased CP digestibility and N-retention and decreased blood urea nitrogen concentration. Replacement of fishmeal 20% with TM larvae 20% in weaned pigs’ diet showed no significant differences in growth performance and digestibility of dry matter (DM), gross energy (GE), and N [26]. Moreover, replacing plasma protein 5% with TM larvae 5% did not affect the growth performance of pigs weaned at d 14 of age for 8 weeks with a reduction of diarrhea rate during 2 to 4 weeks [20]. However, replacement of soybean extraction meal (14.8%) with TM larvae meal (10%) in weaned pigs’ diet reduced ADG of weaned pigs for 4 weeks, whereas it did not affect the ADFI and G:F ratio of pigs [36]. Regarding the results of the previous studies [20,26,36], animal-origin protein sources could be replaced with TM larvae without a reduction in the growth performance of pigs. Furthermore, TM larvae could be included in swine diets up to 20% without detrimental effects on the growth and health of pigs. In gestating sows, increasing level of TM larvae supplementation from 0% to 3% with replacement of fish meal from for late-gestation period (d 75 to 115 of gestation) linearly increased total litter weight without a difference in the number of total born piglets (data unpublished). They also found that TM larvae inclusion in gestation diets did not affect piglet growth during the lactation period and colostrum composition. In lactating sows (data unpublished), supplementation of TM larvae (0% to 3%) with replacement of SBM in lactation diets significantly improved daily feed intake of sows with a numerical increase of litter weight gain during the lactation period (0 to 3 weeks). In addition, supplementation of TM larvae at 10% in weaned pig’s diet showed a weak change of intermediary metabolisms, including gene expression for liver and skeletal muscle tissues and plasma concentration of metabolites in pigs [36]. Furthermore, it did not cause antioxidant stress and activate oxidate stress-sensitive signaling pathways in the liver and gastrocnemius muscle in pigs [37]. Thus, TM larvae could be regarded as a safe protein source for the swine diet in terms of animal health. However, there is a need for further studies for evaluating the nutritional value of TM larvae for growing-finishing pigs with pork quality to utilize the TM larvae as a protein source for the swine diet.

Several studies have evaluated the nutritional value of TM larvae products for ileal digestibility in pigs. Yoo et al [27] reported that growing pigs fed the diet with TM larvae had greater SID of GE, Arg, and Cys compared to pigs fed the diet with fish meal. Also, the TM larvae diet tended to show increased SID of DM, CP, and AAs compared with meat meal, poultry meal, or fish meal diets. Cho et al [28] observed that growing pigs fed the diet containing hydrolysate of TM larvae had greater SID values for DM, CP, Lys, Met, and Thr than pigs fed the diets with fermented poultry by-product and 10% hydrolyzed fish soluble respectively. Although defatted TM larvae meal diet had greater SID values for DM, CP, Lys, Met, Thr, Asp, Gly, and Ala than hydrolyzed fish soluble diet, the SID values of AA (except for Met) had no differ between defatted TM larvae meal and fermented poultry by-products diets. Liu et al [22] indicated that insect meal including TM larvae could improve protein utilization through AA transportation by regulating the sensing gene and the mammalian target of rapamycin (mTOR) signal pathway in the intestinal mucosa. Collectively, TM larvae meal or products are potentially promising protein sources for pigs with an improvement in protein or AA utilization.

### Others

The HF (*Musca domestia*), also known as maggot, has a short life cycle and can be grown in large biomass or organic wastes, hence considered as a sustainable protein source for livestock animals [45]. It contains average 50% CP ranged from 39.2% to 64.0% and 23.5% EE ranged from 20.8% to 25.3% (reviewed by Makinde [45]). Dankwa et al [41] reported that replacement of fish meal (10.8%) with HF larvae meal (10%) had no difference in the growth performance and carcass characteristics of weaned pigs for 10 weeks. Also, Tan et al [23] assessed the AA digestibility values for HF meal that the contents of AA, CP, EE in HF meal were greater than BSF meal and coefficient values of SID for all AA except Met and Cys in HF meal were greater than in BSF meal.

The *Ptectious tenebrifer* larvae, known as mealworm, contained 48.2% of CP, 29.5% of EE, 6.06% of crude fiber, 6.82% of crude ash, 5.4% of total lysine, and 1.5% of total methionine (as-fed basis; [25]). Ao and Kim [25] reported that supplementation of 2% dried mealworm (*Ptecticus tenebrifer*) larvae replacing fishmeal (2%) in weaned pig’s diets had no negative effects on the growth performance of pigs during the overall period (0 to 5 weeks) and had no difference in the apparent total tract digestibility of DM, N, and GE in weaned pigs on 5 weeks.

Superworm (*Zophobas morio*, ZM), known as giant mealworm, belongs to the darkling beetles’ family and is commonly reared as feed for birds and reptiles. The ZM larvae contained 7.4% of total N, 39.3% of EE, 5.3% of crude ash, 2.7% of total lysine, 8% of total methionine, 11.2% of neutral detergent fiber, and 6.4% of acid detergent fiber (DM basis; [46]). The apparent ileal digestibility values of AA and CP for ZM larvae (5%) is similar to those for plasma protein (5%), TM larvae (5%), BSF larvae in early-weaned pigs on d 28 [20]. Also, the apparent ileal digestibility values of DM, AA, and CP in the diet with 5% ZM larvae for weaned pigs on d 56 had no difference compared to those in the diet with 5% plasma protein. Moreover, Liu et al [22] found that supplementation of ZM larvae at 5% modulated the activation of mRNA expression for sodium-coupled neutral AA transporter 2 regulated through the mTOR signal pathway, which is related to intracellular AA transportation.

## CHALLENGES IN THE USE OF INSECT AS A FEED INGREDIENT

Insects have great potential as a feed ingredient for swine diets in nutrient composition, utilization, and functional property. However, some challenges need to be resolved to use the insects in swine feed.

First, standard procedures in the insect production system for rearing, processing, and storage should be established to supply the insects as feed ingredients for pigs by mass production. The standard procedure for the mass production of insects should control the critical hazard point of food safety for consumers and livestock animals [5,47]. In addition to this, developing a processing method to make a novel insect product would give a superior value to the insect [28]. With the increased demand for insects and developed technology for insect production, stable mass production of insects for feed ingredient supply will be made in the near future.

Secondly, the current market price of insects is not competitive with commercially used protein ingredients in swine feed such as SBM and fish meal [14]. Because the insects have been produced to provide feed market for birds and reptiles, the farm or company that produces the insects is relatively smaller than the feed ingredient producers for swine feed. Therefore, it is necessary to set up a large-scale production system to provide the huge amounts of insects stably and improve the production efficiency, leading to a reduction of the market price for the insects. To use the insect as a feed ingredient for swine feed, the price must go down to at least $1.5/kg. The target price of $1.5 is still higher than that of SBM ($0.35/kg; Indexmundi [48]), but the advantages of using the insects as a high-quality protein source will compensate for this gap.

Lastly, further research is needed to identify the nutritional and functional value of insect products for pigs. Previous pig studies have focused on the growth performance, and digestibility of pigs fed the diets with insect products to investigate their potential as a feed ingredient in swine feed. Thus, there is a need to determine the optimal inclusion level of insect products in each growing phase diet regarding growth, reproduction, pork quality, and the health of pigs. In addition, it is necessary to verify the functional effects of insect products (e.g., chitin, bioactive compounds; [15,49]) that can be expected when used in swine diets. Furthermore, there are potential safety issues of insect products for toxic substances [50], antibiotic resistance gene [51], contamination by pathogens or mycotoxins [52,53], and heavy metal accumulation [54].

## CONCLUSION

The insect products used in the previous studies (black soldier fly, yellow mealworm, housefly, etc.) showed great potential as an alternative protein source in swine diets. Many previous studies have been conducted to evaluate the nutritional value of insect products to replace with SBM and fish meal in weaned pig’s diets. In the future, there is a need for research on the optimal inclusion level of insect products in every growth phase of pigs, such as growing-finishing pigs, gilts, and sows, with regards to the pig productivity, pork quality and safety, and consumer’s perception. Additionally, further research on functional properties of insect products or various processing methods for insect products should precede the use of insect products in swine diets to ensure their efficiency and safety.

## Figures and Tables

**Table 1:** Swine research with insect products (black soldier fly, mealworm, housefly, and superworm) by country from 2000 to 2021 years

Country		Insect	Feeding phase	References
Africa	South Africa	BSF larvae (*Hermetia illucens*)	Suckling	[18]
	Kenya		Growing	[19]
	Ghana	HF larvae (*Musca domestica*)	Weaning	[41]
Asia	China	BSF larvae (*Hermetia illucens*)	Growing	[23]
			Finishing	[21]
		HF larvae (*Musca domestica*)	Growing	[23]
			Weaning	[20,22]
		Mealworm (*Tenebrio molitor* larvae)	Weaning	[20,22]
		Superworm (*Zophobas morio* larvae)	Weaning	[20,22]
	South Korea	Mealworm (*Ptecticus tenebrifer* larvae)	Weaning	[25]
		Mealworm (*Tenebrio molitor* larvae)	Weaning	[24,26]
			Growing	[27,28]
Europe	Italy	BSF larvae (*Hermetia illucens*)	Weaning	[32,33]
	Belgium		Weaning	[29]
	Russia		Growing	[30]
	Netherlands		Growing	[34]
			Finishing	[35]
	Germany	BSF larvae (*Hermetia illucens*)	Growing-finishing	[31]
		Mealworm (*Tenebrio molitor* larvae)	Weaning	[36]
			Growing	[37]
North America	Canada	BSF larvae (*Hermetia illucens*)	Weaning	[39]
		BSF larvae (*Hermetia illucens*)	Growing	[38]

BSF, black soldier fly; HF, house fly.

**Table 2:** Nutrient composition (%) of *Hermetia illucens* (black soldier fly, BSF) larvae products (dry matter basis)

%	Full fat BSF larvae meal	Partially defatted BSF larvae meal	Defatted BSF larvae meal
Moisture	2.7–11.6	5.26	3.42–6.1
Crude protein	35.9–48.1	59.0	40.8–60.7
Crude ash	4.98–8.39	8.10	6.8–8.1
Crude fat	36.8–48.1	8.97	7.97–12.28
Crude fiber	6.53	-	-
Neutral detergent fiber	16.18	20.30	13.3
Acid detergent fiber	7.28–8.71	9.18	9.1
Lysine	1.78–2.12	2.81	1.63–2.96
Methionine	0.52–0.85	0.77	0.72–0.77
Threonine	1.20–1.70	2.12	1.05–2.01
Tryptophan	0.45–0.65	3.67	0.71
Calcium	0.55–2.35	8.66	0.58–0.95
Phosphorus	0.53–0.93	13.83	0.76–1.46
Reference	[18,19,21,29,38]	[32]	[29,38]

**Table 3:** Nutrient composition (%) of *Tenebrio molitor* (TM) larvae products (dry matter basis)

%	TM larvae meal	Defatted TM larvae meal	Hydrolysate of TM larvae
Moisture	5.5–5.9	8.5	10.6
Crude protein	43.3–74.0	68.0	58.9
Crude ash	2.9–6.3	8.0	14.9
Crude fat	10.3–34.6	4.0	0.3
Crude fiber	4.6–9.5	-	-
Lysine	1.86–5.42	5.82	10.36
Methionine	0.54–1.34	2.07	3.02
Threonine	1.57–4.04	4.10	3.19
Tryptophan	0.02–0.75	-	-
Calcium	0.04	0.6	0.2
Phosphorus	0.59–0.75	0.5	0.8
Reference	[24,25,27,28,36]	[28]	[28]
